# Production, Characterization, and Flocculation Mechanism of Cation Independent, pH Tolerant, and Thermally Stable Bioflocculant from *Enterobacter* sp. ETH-2

**DOI:** 10.1371/journal.pone.0114591

**Published:** 2014-12-08

**Authors:** Wei Tang, Liyan Song, Dou Li, Jing Qiao, Tiantao Zhao, Heping Zhao

**Affiliations:** 1 Research Center of Environmental Microbiology and Ecology, Chongqing Institute of Green and Intelligent Technology, Chinese Academy of Science, Chongqing, 400714, China; 2 Key Laboratory of Reservoir Aquatic Environment, Chinese Academy of Sciences, Chongqing Institute of Green and Intelligent Technology, Chinese Academy of Sciences, Chongqing, 400714, China; 3 School of Chemistry and Chemical Engineering, Chongqing University of Technology, Chongqing, 400054, China; 4 Ministry of Education, Key Lab of Environmental Remediation and Ecosystem Health, College of Environmental and Resource Science, Zhejiang University, Hangzhou, 310058, China; Montana State University, United States of America

## Abstract

Synthetic high polymer flocculants, frequently utilized for flocculating efficiency and low cost, recently have been discovered as producing increased risk to human health and the environment. Development of a more efficient and environmentally sound alternative flocculant agent is investigated in this paper. Bioflocculants are produced by microorganisms and may exhibit a high rate of flocculation activity. The bioflocculant ETH-2, with high flocculating activity (2849 mg Kaolin particle/mg ETH-2), produced by strain *Enterobacter* sp. isolated from activated sludge, was systematically investigated with regard to its production, characterization, and flocculation mechanism. Analyses of microscopic observation, zeta potential and ETH-2 structure demonstrates the bridging mechanism, as opposed to charge neutralization, was responsible for flocculation of the ETH-2. ETH-2 retains high molecular weight (603 to 1820 kDa) and multi-functional groups (hydroxyl, amide and carboxyl) that contributed to flocculation. Polysaccharides mainly composed of mannose, glucose, and galactose, with a molar ratio of 1∶2.9∶9.8 were identified as the active constituents in bioflocculant. The structure of the long backbone with active sites of polysaccharides was determined as a primary basis for the high flocculation activity. Bioflocculant ETH-2 is cation independent, pH tolerant, and thermally stable, suggesting a potential fit for industrial application.

## Introduction

Flocculating agents, widely used in industrial processes such as wastewater treatment, are classified into three groups i.e., inorganic flocculants such as aluminium sulphate; organic synthetic high polymer flocculants such as polyacrylamide (PAM); natural flocculants or bioflocculant such as microbial flocculant [1], [2]. Synthetic high polymer flocculants have been most frequently utilized due to their flocculating efficiency and low cost, however, there is increasing evidence of risk to human health and the environment [1]–[3]. Acrylamide monomer, for example, is derived from PAM and acts as a strong carcinogen and neurotoxin to humans.

Bioflocculant research seeks the benefits of a treatment with high production rate and biodegradable, innocuous waste products while solving the challenges of its tendency for low flocculation activity and high cost. Varying types of microorganisms, including bacteria [4]–[10], fungi [11], [12] and algae [13], [14], have been isolated from soil and activated sludge, and shown to produce bioflocculant. Recent studies have more specifically focused on various bioflocculant producing bacteria including; (*Enterobacter aerogenes* [4], *Halomonas* sp. [5], *Chryseobacterium daeguense* [6], *Klebsiella pneumoniae* [7], *Paenibacillus* sp. [8], *Corynebacterium glutamicum* [9], *Bacillus licheniformis* [10], et al.).

Bioflocculant research has focused on the isolation of bioflocculant-producing microorganisms and their production, whereas flocculation mechanisms and active constituents of bioflocculation are generally undetermined; this is especially true for cation independent bioflocculant. Active constituents within bioflocculant structure must be understood to fully comprehend flocculation functions. Colorimetric methods for the quantification of bioflocculant chemicals composition, Gel Permeation Chromatography (GPC) for Molecular Weight (MW) characterization, Fourier Transform Infrared Spectroscopy (FTIR) and X-ray Photoelectron Spectroscopy (XPS) for flocculation functional group identification have been utilized in limited studies to elucidate the composition and structure of bioflocculant [10]; however, the relationship between their structure and flocculation activity remains unclear.

In this study we isolated a microorganism, ETH-2, from activated sludge and identified it as *Enterobacter* sp. (*E*. sp.), which secretes bioflocculant. High flocculation activity to kaolin suspension without the aid of cations occurs with this bioflocculant. The production culture medium composition of *E*. sp. ETH-2 was optimized and the main factors influencing flocculation efficiency (pH, temperature, dosage, and cation) were then investigated. Bioflocculant structure was characterized by combining the chemicals analysis, GC-MS, GPC, FTIR, and XPS with the flocculation activity. The flocculation mechanism was then proposed according to the results.

## Materials and Methods

### Ethic Statement

Activated sludge used in this study was obtained from the secondary settling tank of Tang Jia Qiao municipal wastewater treatment plant (WWTP) in Chongqing, China. All necessary permits for collection of the sludge from this system were granted by Tang Jia Qiao Municipal WWTP and Chongqing Municipal Construction Bureau. All field work has been conducted according to the relevant national guidelines.

### Isolation of flocculant-producing strain ETH-2 of *Enterobacter* sp

#### Isolation of strain

Activated sludge samples were collected in 1L sterilized Nalgene HDPE big mouth sample bottles (Fisher Scientific, USA) and stored on ice during transport back to the laboratory with immediate treatment upon arrival at the lab. Following 10 fold series dilution, 100 ul dilutions were planted on Luria-Bertani (LB) agar plates and cultured at 30°C overnight. Strains with unique colony morphologies were selected and inoculated into 250 ml flasks containing 100 ml flocculation selecting mediums for 3 days at 30°C with 180 rpm shaking. One ml culture was utilized for flocculation activity test assay. Fifty-four strains were isolated. Within them, ETH-2 strain demonstrated the highest flocculating activity and was selected for further study. The SEM image of the strain was obtained by a scanning electron microscope (Helios, NanoLab 600i, FEI). Strain ETH-2 has been deposited into the China Center for Type Culture Collection (CCTCC) (Wuhan, China) (accession number CCTCC M 2013042).

The LB agar contains (per liter): tryptone, 10 g; yeast extract, 5 g; and NaCl, 10 g; agar, 15 g. The flocculation selecting medium contains (per liter): Glucose 10 g; KH_2_PO_4_ 2 g; K_2_HPO_4_ 5 g; MgSO_4_·7H_2_O 0.2 g; NaCl 0.1 g; urea 0.5 g; yeast extract 0.5 g. After optimization of carbon and nitrogen source (Materials S1 and Figure S1 in File S1), the culture media was slightly modified as containing (per liter): glucose, 10 g; NaNO_3_, 1 g; KH_2_PO_4_, 2 g; K_2_HPO_4_, 5 g; NaCl, 0.1 g; MgSO_4_·7H_2_O, 0.2 g. The initial pH of all media was adjusted to 7.3±0.1 with NaOH (1 M) and HCl (0.5 M).

#### Identification of strain

DNA was extracted, according to vendor protocol, utilizing the TIA Namp Bacteria DNA Kit (TianGen, China). The 16S rRNA gene was amplified utilizing bacterial universal primers 27F (5′-AGAGTTTGATCCTGGCTCAG-3′) and 1492R (5′-GGTTACCTTGTTACGACTT-3′) under the following conditions: Initial denaturation at 95°C for 10 min, followed by 30 cycles (30 s at 95°C, 30 s at 55°C, and 1 min 30 s at 72°C) and a final 10 min extension at 72°C. The PCR production was then purified utilizing the QIAquick PCR Purification Kit (Qiagen, USA), cloned into pMD19-T vector (Takara, China), and then sequenced. The obtained 16S rRNA gene sequence was assembled with Seqman II 5.0 (DNASTAR) [15] and analyzed using the BLAST program. Phylogenetic tree construction was followed as previous described [16]. The 16S rRNA gene sequence has been deposited into the GenBank with accession number KF739069.

### Purification and Characterization of ETH-2 Bioflocculant

#### Extraction and Purification of ETH-2 Bioflocculant

ETH-2 was inoculated into a 200 ml optimization medium and cultivated at 30°C for 24 h at 180 rpm and the cultured broth was harvested for bioflocculant production extraction. Comparisons of flocculation extraction efficiency through varying purification approaches were attained. Heating at 80°C demonstrated the highest extraction efficiency (Table S1 in File S1) and was used for bioflocculant extraction as previously described [17]. Briefly, the cultured broth was centrifuged at 12,000 rpm for 5 min and then washed three times with 0.9% NaCl. The suspended pellets were dissolved into 0.1 volume 0.9% NaCl and heated at 80°C for 15 min.

Bioflocculant purification was performed using a minor modification of the method described [10], [18]. After natural cooling, the heating extracted cell-free supernatant was obtained by centrifugation at 12,000 rpm for 10 min and filtered with 0.22 µm membrane. Cold ethanol was added to the supernatant and left overnight at 4°C. The precipitate was collected by centrifugation at 12,000 rpm for 10 min and dissolved in ultrapure water and then dialyzed with a molecular weight cutoff: 8000–14400 Da (36 MM, Biosharp, USA). Cetyltrimethyl ammonium bromide (CTAB) (2%, wt.%) was added to the solution with stirring at 100 rpm. After 3 h, the CTAB-bioflocculant complex precipitate was collected by centrifugation and then dissolved in 0.5 M NaCl solution. Two volumes of cold ethanol were added to solution to obtain the precipitate, and the precipitate was washed several times with ethanol. The precipitate was dissolved in ultrapure water and dialyzed against ultrapure water overnight. Finally, the purified bioflocculant, designated as ETH2, was obtained and lyophilized for further experiments.

#### Characterization of ETH-2 Bioflocculant

Determination of total sugar was completed utilizing the phenol-sulfuric acid method with glucose as the standard solution [4], [10]. Protein was determined by applying the Bradford method with the bovine serum albumin (TianGen, China) as the standard. Analysis of monosaccharide composition was determined by GC-MS with the following procedure: 10 mg lyophilized ETH-2 was hydrolyzed with 2 ml of 1 M H_2_SO_4_ in 100°Coil bath for 4 hours in a sealed glass tube. The hydrolysate was then centrifuged at 8000 rpm for 5 min after its pH was neutralized to 7.0 with BaCO_3_. The supernatant was filtered with 0.22 µm hydrophilic filter membrane (JinTeng, China) and the filtrate collected for lyophilized (Virtis, BT4KXL, USA). The lyophilized powder reacted with 10 mg hydroxylamine hydrochloride and 0.5 ml pyridine in 90°Cwater bath for 30 min and then was acetylated with 0.5 ml acetic anhydride in 90°C water bath for another 30 min. The acetylated aldononitrile of ETH-2 were analyzed on Agilent 7890A-5977 GC/MS operated in the EI mode and a HP-5MS fused silica capillary column (30 m×0.32 mm×0.25 mm). Helium was used as carrier gas with 1 ml/min flow rate. Temperatures of injector and detector were set at 300°C and 260°C, respectively, and the initial column temperature was 130°C for 5 min, increased to 240°C at a rate of 4°C/min and held for 5 min. Sugar identification was performed by comparison with reference sugars. Relative molar proportions were then calculated by the area normalization method.

The molecular weight of ETH-2 was analyzed by using Lc-10ADVP Gel Permeation Chromatography (GPC) equipped with RID-10A detector (Shimadzu, Japan) and TSK G4000PWxl columns (Shimadzu, Japan) at an optimal operation temperature 40°C. The column was calibrated by standard dextrans. DDI water was used as the mobile phase with 0.5 ml/min flow rate. Prior to injection, the sample was filtrated utilizing a 0.45 µm filter. Number and weight-average molecular weight served to characterize the bioflocculant MWs [19].

The element of ETH-2was analyzed by X-Ray Photoelectron Spectroscopy (XPS) (XSAM800, Kratos, UK) under FAT mode with a vacuum degree of 5×10^−7^ Pa, a power of 12 kv and electric current of 12 mA. The laser source was aluminum target and the 284.8 eV was used for correction.

The Fourier Transorm Infrared Spectrometer (IR) (ThermoFisher, Nicolet 6700, USA) was used for the detection of functional groups of ETH-2 and IR of ETH-2 sample was measured from 400–4000 cm^−1^.

### Flocculation of Kaolin by ETH-2 Bioflocculant

#### Flocculation dynamics

Flocculation dynamics of cell and produced biofloculant was measured according to the used kaolin suspension method. One ml agent was mixed into 50 ml of 4 g/L kaolin suspension in a 50 ml graduated cylinder covered with clean film. The test cylinder was gently shaken and settled at room temperature. At prescribed time, 3 ml of supernatant was carefully removed from the upper layer of solution and measured at a 550 nm absorbance. Control experiment was also conducted without flocculation agents.

##### Flocculation impact factors

Effects of impact factors (dosages, cations, pH and temperature) were investigated to study the flocculation activity of the ETH-2. Purified ETH-2 bioflocculant was dissolved in ultrapure water to get a 0.066 mg/ml concentration. A series volume (0.05, 0.1, 0.2, 0.5, 1, 2, 5, 10, 20 and 50 ml) of ETH-2 solution, equal to 0.003, 0.007, 0.013, 0.033, 0.066, 0.132, 0.330, 0.660, 1.320, and 3.300 mg ETH-2, were mixed into Kaolin suspension (200 mg Kaolin particles in 50 ml solution) to test the dosage impact. Cations effects were tested by adding varying cations, 1 milliliter/each (NaCl, KCl, CaCl_2_, MgCl_2_, AlCl_3_, FeCl_3_, concentration is 0.09 M) solution to the Kaolin suspension system. pH values of Kaolin clay solution were then adjusted to 3.0, 4.0, 5.0, 6.0, 7.0, 8.0, 9.0 and 10.0 respectively, with HCl (0.5 M) and NaOH (0.5 M), and then mixed with ETH-2 solution to test pH influence. Thermal stability of ETH-2 evaluation was determined by measuring the flocculation efficiency after ETH-2 was incubated at various temperatures (40, 50, 60, 70, 80, 90, 100°C) for 30 minutes [20].

##### Flocculation mechanism

The zeta potentials of ETH-2, Kaolin clay suspension, and mixture of ETH-2 and Kaolin clay were measured by a zeta potential analyzer (Malvern, UK) to test flocculation mechanism (bridging or charge neutralization).

### Flocculation activity evaluation

Flocculation activity was measured according to the previously described kaolin suspension method [2], [21], [22]. One ml of agent was mixed with 50 ml of 4 g/L kaolin suspension in a 50 ml graduated cylinder covered by clean film. The test cylinder was gently shaken and settled for 5 min at room temperature. Three ml of supernatant were carefully removed from the upper layer of solution and measured at a 550 nm absorbance (TU-1901, Persee, China). A separate control experiment was conducted without flocculation agents. Flocculation activity was calculated according to the following equation: 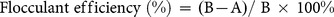



Where A is the OD_550_ of sample, while B is the OD_550_ of control.

## Results and Discussion

### Isolation of flocculant-producing strain ETH-2 of *Enterobacter* sp

**Figure 1:**
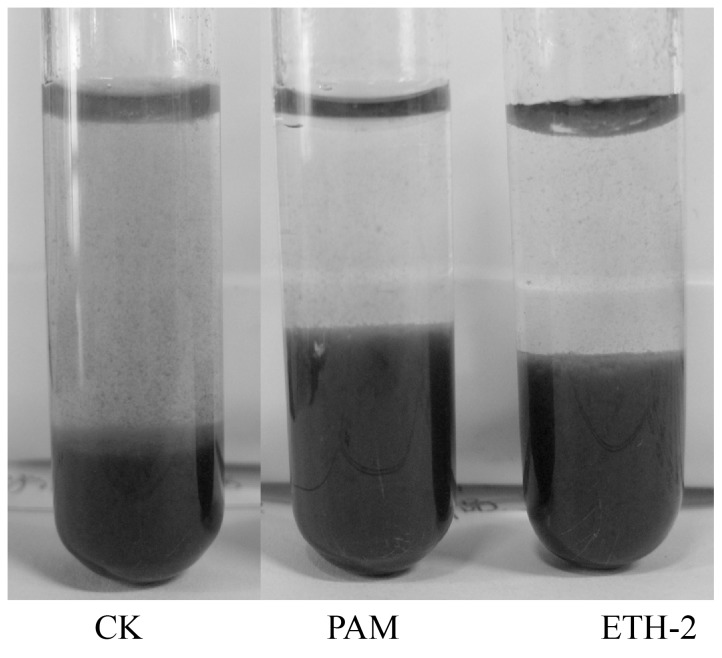
Comparison of flocculation ability by ETH-2 and PAM for wine process wastewater. Three hundred µl ETH-2 strain culture broth (OD_600_ = 1.8) and 0.1% PAM were added into 5 ml wine process wastewater (COD: 4299.0 mg/L; pH = 4.0; SS = 315.0 mg/L), respectively. The cylinder was gently shaken, settled at room temperature for 5 min, and measured the OD_550_.

**Figure 2:**
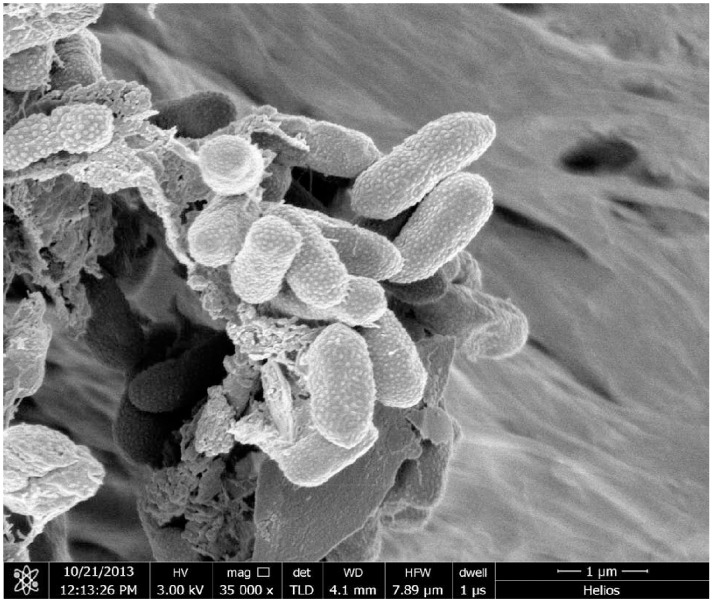
SEM image of *Enterobacter* sp. ETH-2.

**Figure 3:**
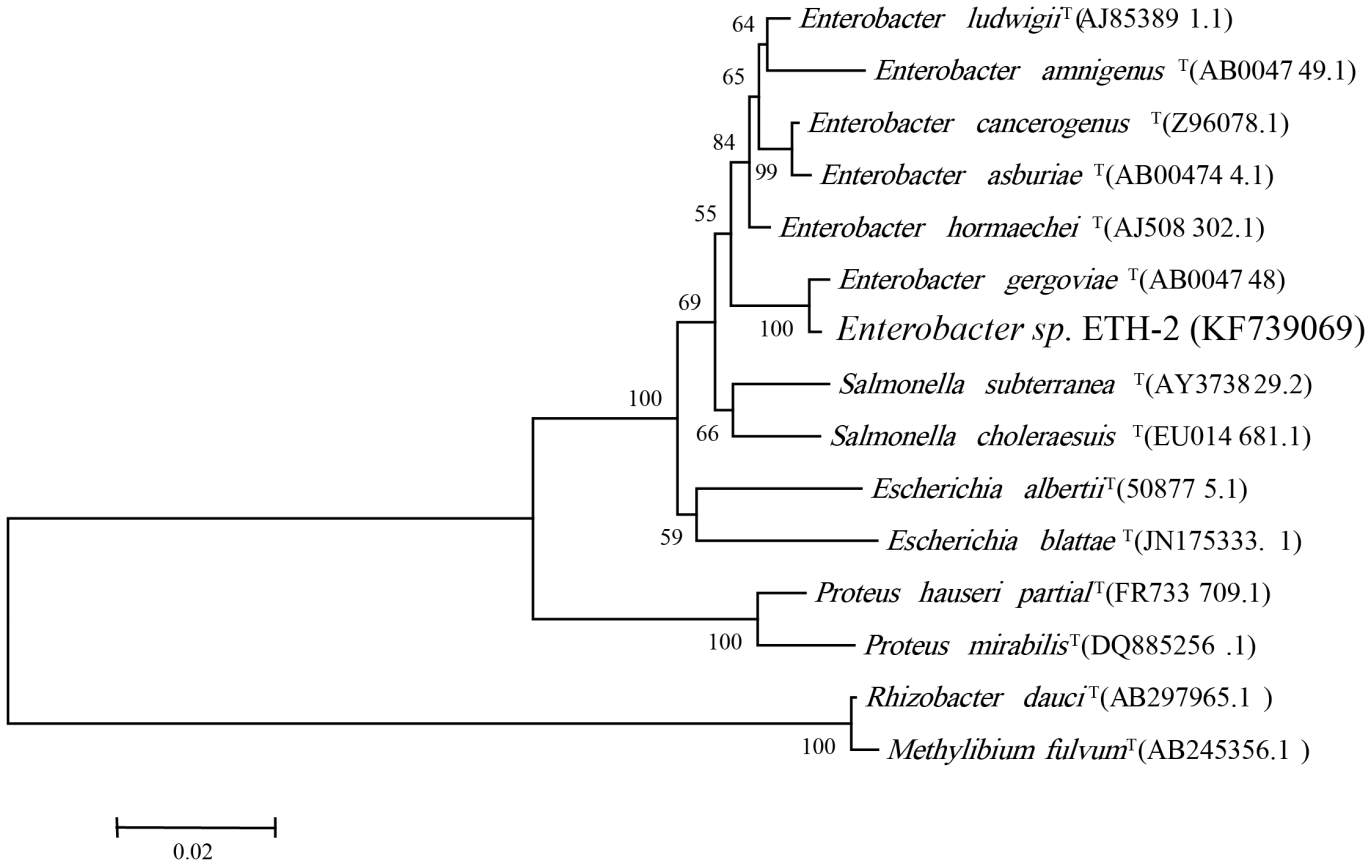
Phylogenetic tree based on 16S rRNA sequences (Bar: sequence dissimilarity of 0.02%).

We compared the flocculation efficiency of ETH-2 strain culture broth (OD_600_ = 1.8) and PAM (0.1%, w/v %) in the test of flocculation of wine process wastewater and the former showed higher flocculation efficiency (Figure 1). Figure 2 shows the morphological analysis of the strain ETH-2. The strain formed rounded, cream-colored, smooth colonies on agar culture media, while the SEM image indicates the strain was short rod-shaped, flagellated, motile aerobe and had convex. The phylogenetic identity of the strain ETH-2 by 16S rDNA sequencing revealed that it was most closely to *Enterobacter gergoviae* (98% similarity) (Figure 3). Although in the past, several bioflocculant producing bacteria have been noted, few studies report bioflocculant production with *Enterobacter* sp. as the source. Currently, just four strains affiliated with *Enterobacter*, including *Enterobacter* sp. BY-29 [23] , *Enterobacter cloacae* WD7 [20], *Enterobacter* sp. EP3 [24] and *Enterobacter aerogenes* [4], have been isolated and their producing bioflocculant characterized.

### Purification and Characterization of ETH-2 Bioflocculant

#### Characterization of the purified ETH-2

**Figure 4:**
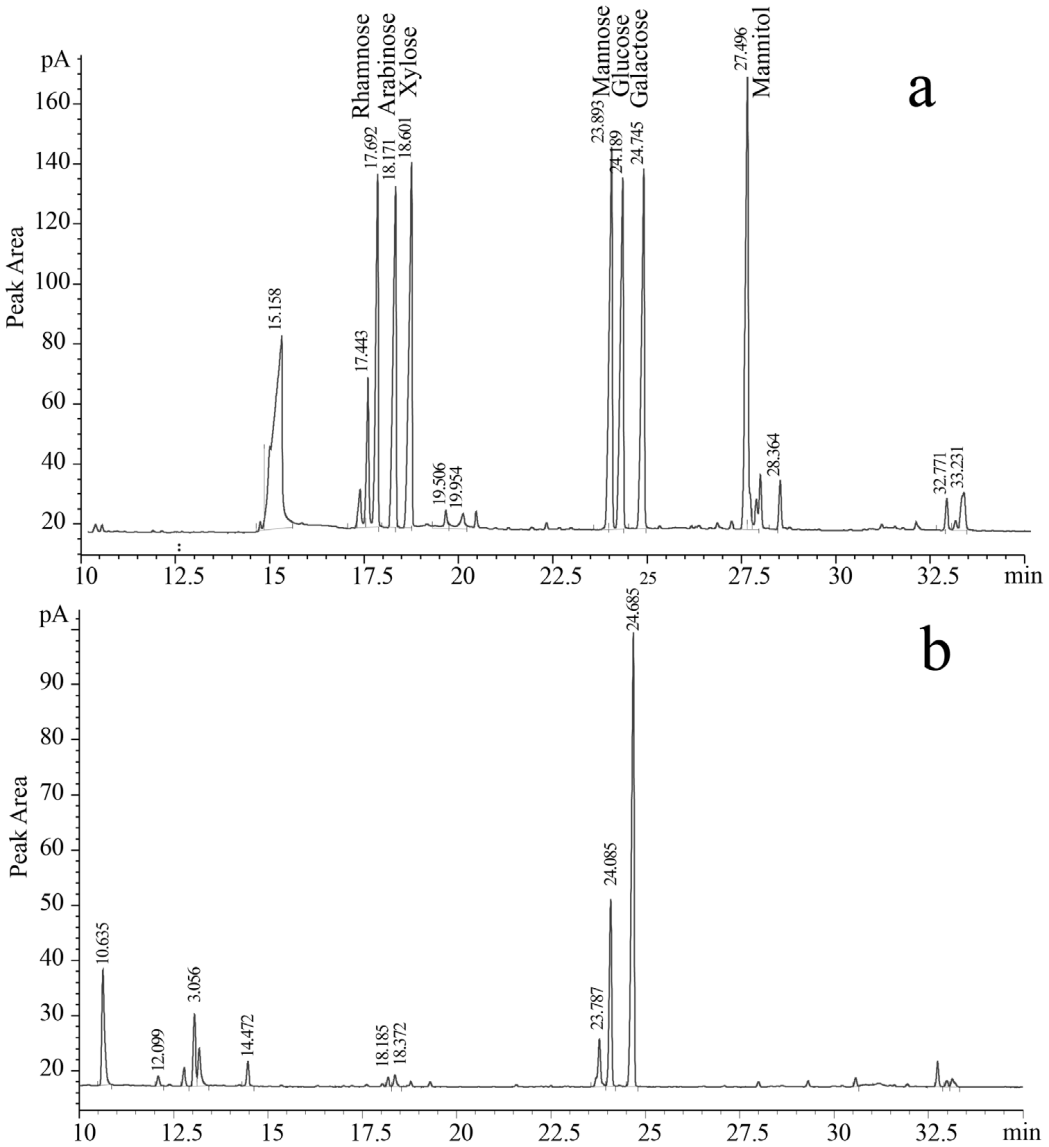
Gas chromatogram of standard monosaccharide (a) and alditol acetate derivatives from ETH-2 (b).

A total of 65.6 mg of purified ETH-2 was recovered from 1 L of fermentation broth. The total sugar and total protein content of ETH-2 was 91.7% and 1.8%, respectively, indicating a major polysaccharides component. Comparison of retention time and corresponding mass spectra with known standards (Figure 4a) was achieved through examination and identification of the alditol acetate derivatives from monosaccharide by GC-MS. Three major carbohydrate peaks at 23.79, 24.09, and 24.69 min were depicted on a gas chromatogram (Figure 4b), which were attributed to mannose, glucose, and galactose, respectively, with a molar ratio of 1∶2.9∶9.8.

**Figure 5:**
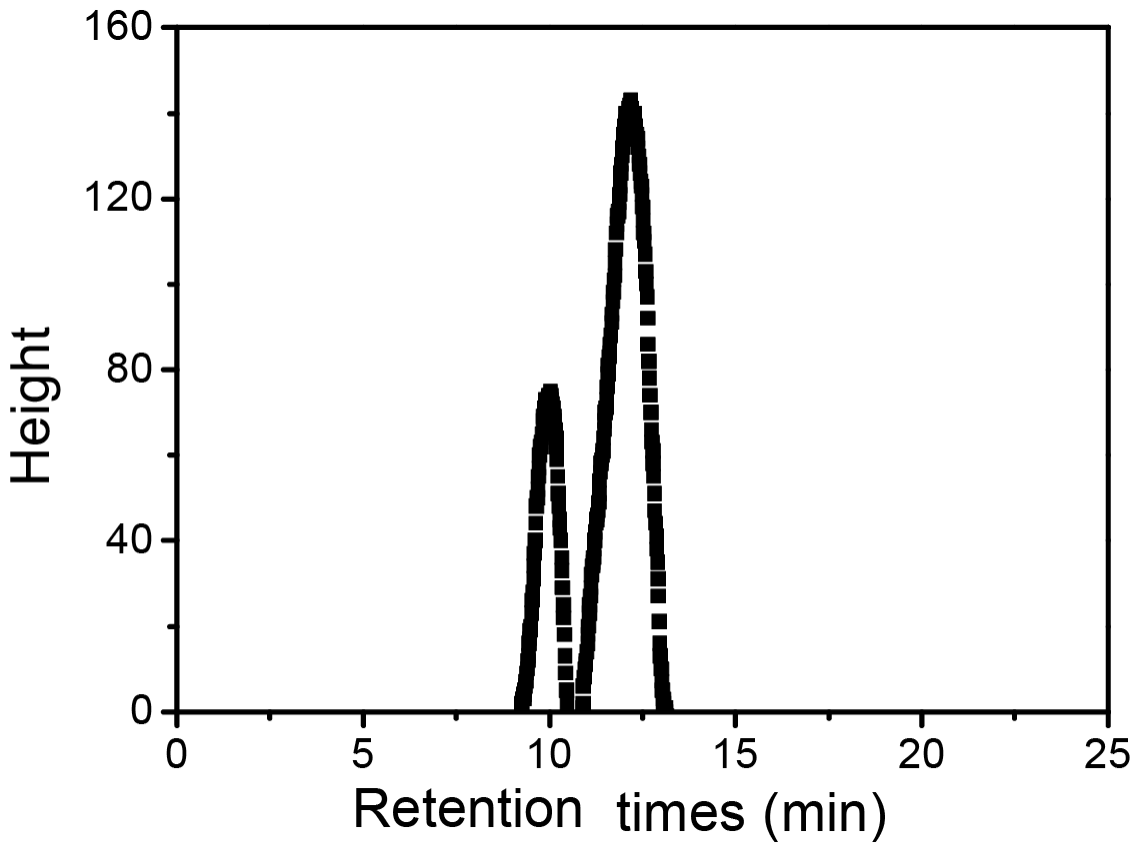
GPC chromatogram of ETH-2.

RI chromatograms with two retention times (10.03 and 12.18 minutes) of ETH-2 are represented in Figure 5. The apparent weight and number averaged MWs (*M*_w_ and *M*_n_) of the two constituents are listed in Table S2 in File S1. The MWs of the main constituents in ETH-2 ranges from 603to 1820 kDa, which is within the high MWs range, demonstrating the high flocculation ability of ETH-2. High MW bioflocculant possess more adsorption points for bridging so greater flocs are obtained [1], [25]. The reported efficient bioflocculant usually has high MWs, ranging from 10^2^ to 10^3^ kDa. For example, *Bacillus megaterium* TF10 produced 1.0–2.5×10^3^ kDa bioflocculant [26] and *Bacillus licheniformis* yield 1.8×10^3^ kDa bioflocculant [10].

**Figure 6:**
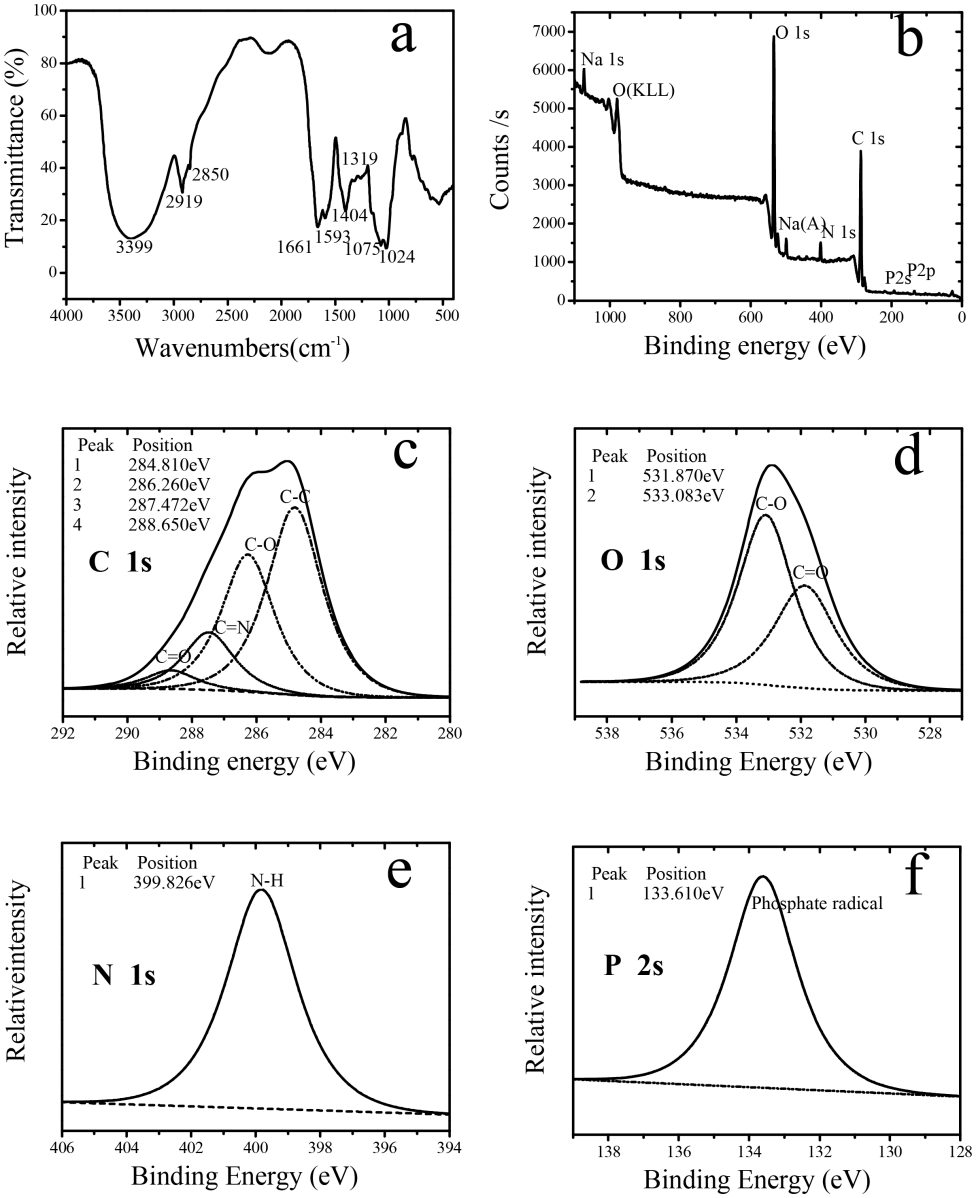
FTIR spectra of ETH-2 (a), X-ray photoelectron spectra of ETH-2 (b) and high resolution 1 s XPS spectra of C, O,N, and P from ETH-2 are shown in (c), (d), (e) and (f), respectively.

The elemental analysis of ETH-2 is indicated in Figure 6. Peaks of 1 s core level of C, O, and N are clearly visible, accounting for 64.7%, 28.6%, and 4.6%, mass fractions, respectively. A peak of 2 p core level of P is detected, presenting a small amount of mass fraction, 1.0%. XPS also detected lower (1.0% mass fraction) 1 s core level of Na, probably due to sodium contamination. High resolution scans of C 1s, O 1s, N1s, and P 2p were deconvoluted to obtain the corresponding functional groups. Total four C (1s) (Figure 6c), two O (1s) (Figure 6d), one N (1s) (Figure 6e), one P (2p) (Figure 6f), and one Na (1s) were observed with the assignment and quantification of these functional groups listed in Table S3 in File S1. The C 1s peak was resolved into four component peaks. The peaks at 284.8 eV are associated with C-(C,H) of lipids or amino acid side chains, accounting for the largest percentage in the spectral band (31.3%). The peak at 286.2 eV, which is attributed to C-(O) from alcohol, ether amine, or amide, also presents a large part (21.9%). The peaks at 287.4 eV are associated with O = CO, as in carboxylate, carbonyl, amide, acetal, or hemiacetal respectively, account for 8.7%. The peaks at 288.6 eV associated with C = O, as in carboxyl or ester, are much lower (2.8%). The O1s peak is decomposed into two peaks. The O1s peak at 531.9 eV (11.5%) is mainly attributed to the C = O, as in carboxylate, carbonyl, ester, or amide. The second O1s peak at 533.1 eV (17.1%) is attributed to C-O, as in alcohols, hemiacetal, or acetal groups. Single peak is detected in N 1s and P 2p, respectively. The peak at 399.9 eV is attributed to nonprotonated nitrogen (Nnonpr, 4.58%) from amines and amides. The peak at 133.6 eV is attributed to phosphate (0.09%).

As shown in Figure 6a, the intense absorption peak at 3,399 cm^−1^ (characteristic of a hydroxyl group) could be caused by the vibration of -OH or -NH in the sugar ring of polysaccharides. Two small stretching bands at 2,919 cm^−1^ and 2,850 cm^−1^ indicated  = CH_2_ of carbohydrate. Predominantly assigned to C = O stretching associated with proteins, is the band at 1661 cm^−1^, while the band at 1593 cm^−1^ originates from N-H bending and C-N stretching vibrations in -CO-NH of proteins. The band at 1404cm^−1^ is attributed to C = O symmetric stretching of –COO groups. Assigned to C-H stretches associated with lipids, is the very small band at 1319 cm^−1^. Two weak peaks at 1075 cm^−1^ and 1024 cm^−1^ indicate C-O is also the characteristic absorption peak of polysaccharides.

The Infrared Spectrum Analysis revealed characteristic peaks for polysaccharides, protein and other materials such as lipids, and demonstrated the presence of the functional groups: hydroxyl (-OH), amide (-CO-NH) and carboxyl (-COO-). FTIR and XPS analysis demonstrates the presence of hydroxyl (-OH), amide (-CO-NH), and carboxyl (-COO^−^) in ETH-2. These function groups are preferred for the flocculation process for purposes similar to observations made in other bioflocculants [3], [25]–[27]. Two major roles in flocculation are important attributes of these function groups: hydrophilicity characteristics are utilized to extend the polymer chain [25]; and the groups span the gap between articles to adsorbs particles [3], [26].

Glycoproteins [28], [29], lipids [30] and especially polysaccharides [3], [10], [11], [31] and proteins [32], [33], have been found to be the key constituents of bioflocculants. For instance, *Proteus mirabilis* [3], *Bacillus licheniformis* [10], *Aspergillus flavus* [11], and *Pullularia Pullulans* [31] produced polysaccharides based bioflocculant, while *Nocardia Amarae* [33] and *Pseudomonas* sp. A-99 [32] produced protein based bioflocculant. Polysaccharides bioflocculants composed of mannose, glucose, and galactose are the key constituents in this study and are the active constituents of ETH-2 (as opposed to protein) as further demonstrated by the thermal stability qualities of ETH-2.

### Flocculation of Kaolin by ETH-2 Bioflocculant

#### Flocculation behavior

**Figure 7:**
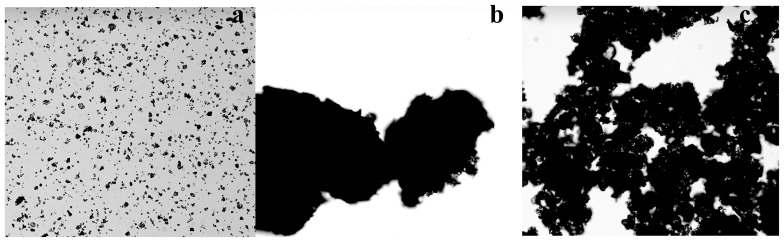
Microscopy imagine of Kaolin clay (a), microscopy imagine of Kaolin clay flocculated by *Entercbacter* sp. ETH-2 (b), and microscopy imagine of Kaolin clay flocculated by ETH-2 (c), respectively.

**Figure 8:**
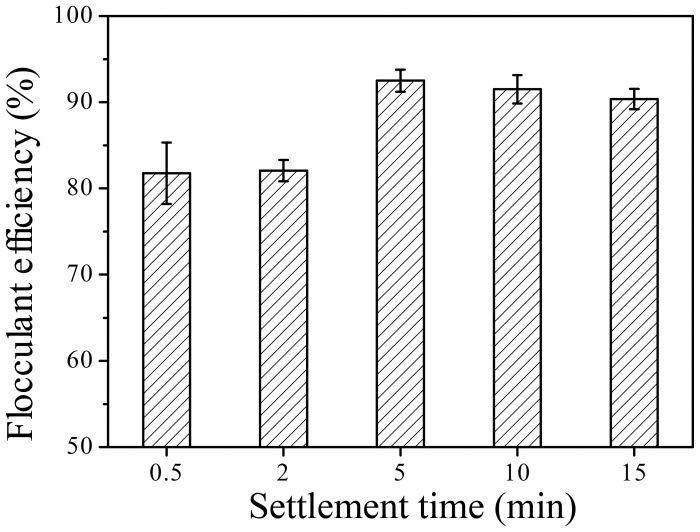
Flocculation dynamics of ETH-2.

Between 200–300 µm flocs were formed by the ETH-2 strain when tested for kaolin clay flocculation (Figure 7b), indicating its advantage on flocculation. The purified bioflocculant ETH-2 also formed 100–200 µm flocs during kaolin clay flocculation testing (Figure 7c), larger than flocs formed by *Bacillus megaterium* TF10 (20–30 µm) [26]. The flocculation dynamics indicated that flocculation efficiency of ETH-2 reached 81.8% at 0.5 min, peaked (92.5%) at 5.0 min, and kept high flocculation efficiency (above 90.4%) within 15 min (Figure 8), suggesting that ETH-2 has quick and high flocculation ability.

Table 1 provides the comparison of Extracellular Polymeric Substances (EPS) based bioflocculants yields by different strains. The flocculation efficiency of ETH-2 is higher than the other reported bioflocculants. For instance, 1.3 mg/L ETH-2 could obtain 94.0% flocculation efficiency, adversely, 30.2 mg/L *Bacillus megaterium* TF10 gained 95.0% flocculation efficiency [26].

**Table 1:** The comparisons of different bioflocculants.

Microorganism	Source	Optimized dose (mg/L)	Efficiency	Cation	References
*Enterobacter aerogenes*	Cell-free supernatant of broth	90	ND	Zn^2+^(30 mM)	[4]
*Enterobacter cloacae* WD7	Cell-free supernatant of broth	2	ND	CaCl_2_(40 mM)	[20]
*Enterobacter sp.* BY-29	The cell surface	40	ND	Fe^3+^(0.2 mM)	[23]
*Enterobacter sp.* EP3	Cell-free supernatant of broth	2	96%	CaCl_2_(8 mM)	[24]
*Bacillus licheniformis*	Cell-free supernatant of broth	5.8	>90%	CaCl_2_(5.14 mM)	[10]
*Bacillus* sp. F19	Cell-free supernatant of broth	2	97%	No cation	[41]
*Bacillus mojavensis* 32A	Cell-free supernatant of broth	10	92.2%	CaCl_2_(1.35 mM)	[19]
*Halomonas* sp. V3a’	Cell-free supernatant of broth	4	96.9%	CaCl_2_(11.25 mM)	[5]
*Bacillus circulans*	Cell-free supernatant of broth	2	99%	CaCl_2_(9 mM)	[27]
*Bacillus firmus*	Most (*>*85%) was in culture broth. Little localized on the cell surface.	4	>60*	CaCl_2_(6.8 mM)	[34]
*Bacillus megaterium* TF10	The cell surface	30.2	95.5%	CaCl_2_(5.6 mM)	[26]
*Entercbacter* sp. ETH-2	The cell surface	1.3	94.0%	No cation	This research

ND: data not shown.

#### Flocculation Impact factors

**Figure 9:**
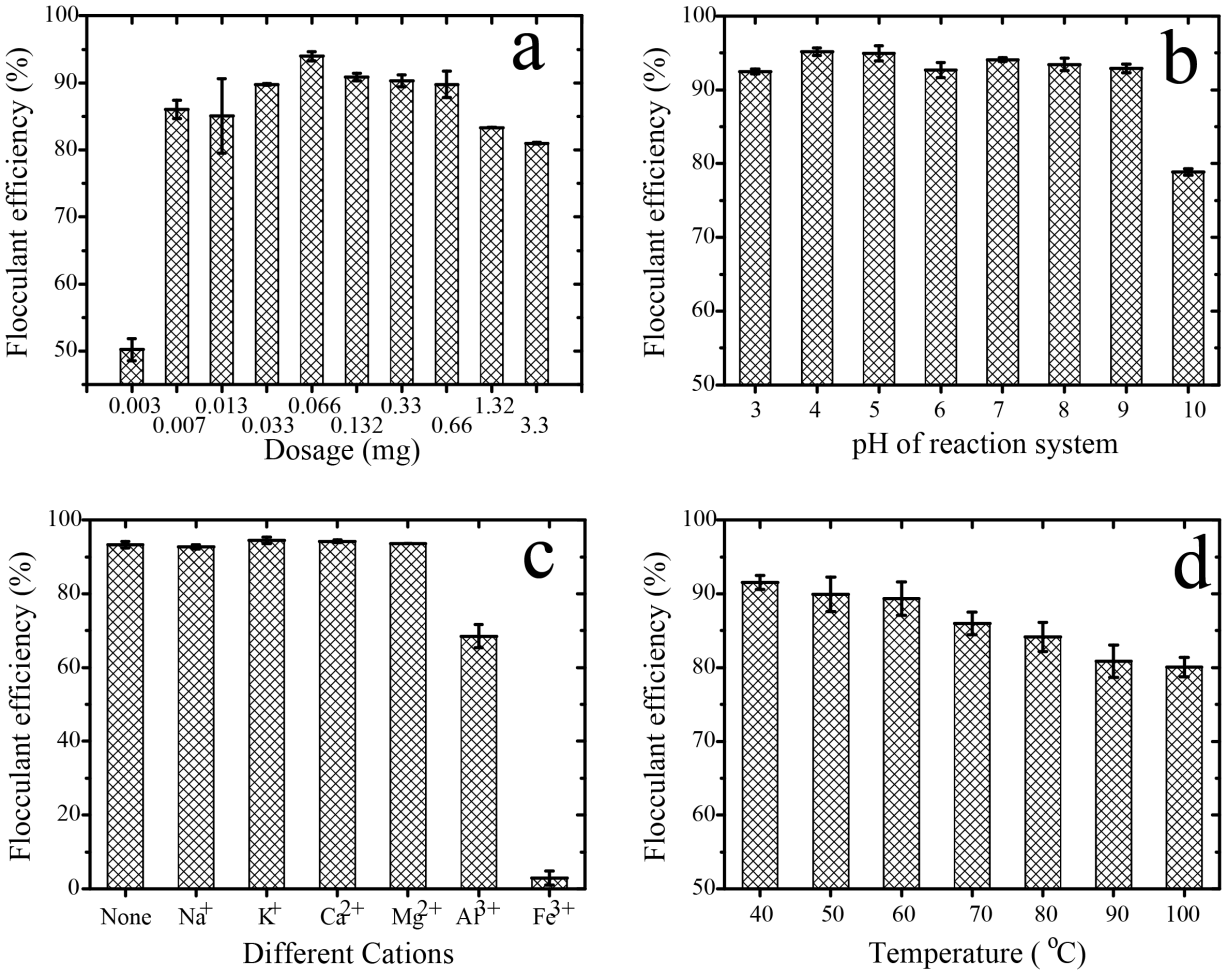
Effect of dosage (a), pH (b), cation (c), and temperature (d) on the flocculating efficiency of ETH-2.

Dosage effect of purified bioflocculant on flocculant efficiency was shown in Figure 9a. Flocculation efficiency of ETH-2 increased from 50.2% to 94.0% with the addition of bioflocculant dose at the range of 0.003 mg to 0.066 mg ETH-2, decreasing to 83.3% with further dose addition to 3.3 mg, indicating 0.066 mg ETH-2 provides optimum flocculation efficiency for 200 mg Kaolin particle. Correspondingly, the maximum adsorption capacity of ETH-2 is calculated as 2849 mg Kaolin particle/mg ETH-2. Similar to other flocculating agents, more or less dose would deteriorate flocculation [27], [34]. Less dose of ETH-2 caused inadequate bioflocculant molecules to adsorb the suspended Kaolin clay particles and to bridge between them. Adversely, more dose of ETH-2 inhibited flocs growth due to the stronger repulsion force between them [18], [26].

Figure 9b illustrates the effect of pH on the reaction system for flocculant efficiency. Flocculation efficiency of ETH-2 kept stable (92.5%–95.2%) at a wide pH range of 3 to 9 and decreased at pH 10(78.9%). Deteriorating flocculation efficiency of ETH-2 with increasing pH possibly results from high alkaline degradation effects on polysaccharide, causing molecular rearrangement of its residue or fragmentation of the polysaccharide chain [35]. The stability of ETH-2 at the range of pH 3-9 was the same as that of the bioflocculant from *Enterobacter cloacae* WD7 [20], but has wider pH range than that of *Gyrodinium impudicum* KG03 that was stable at range of pH 3–6 [14].

Figure 9c demonstrates the effect of different cations on flocculant efficiency. ETH-2 flocculation efficiency is not impacted by Na^+^, K^+^, Ca^2+^ and Mg^2+^, but dramatically influenced by Al^3+^ and Fe^3+^, especially by Fe^3+^, indicating that bioflocculant of ETH-2 is cation independent. Limited cation independent bioflocculants such as *Chryseobacterium daeguense* W6 [36] and *Klebsiella pneumonia* [37] have been reported. Cations stimulate flocculation by neutralization and stabilization of residual negative charges of carboxyl groups in polysaccharide by forming bridges between kaolin particles and consequently improving the flocculation ability for most previous reported bioflocculants [23]. For instance, bioflocculant from *Enterobacter* sp. required Al^3+^, Fe^3+^ and Ca^2+^ for high flocculating activity [23]. Calcium chloride produced a synergistic effect on kaolin flocculation for bioflocculant produced by *Enterobacter cloacae* WD7 [20] and *Enterobacter* sp. EP3 [24]. However, trivalent cations possibly alter the surface charge of kaolin particles and cover the adsorb sites [38]. The competition of the positively charged particles and less adsorb sites induce the low flocculating activity, explaining the flocculation activity reduction in the presence of Al^3+^ and Fe^3+^.

Figure 9d illustrates thermal stability of purified bioflocculant. With the rising of temperature in the range of 40–100°C, the flocculant efficiency reduced from 91.5% to 80.1%, revealing that ETH-2 is relatively stable at this range. Flocculant activity of bioflocculant with a key component of protein is strongly influenced by high temperature [33]. High temperature can destroy the dimensional structure and cause the denaturation of protein, leading to loss of flocculation activity. Adversely, the flocculation activity of polysaccharides bioflocculant cannot be reduced or less reduced under high temperature [27], [39] due to the heat resistance of polysaccharides. Some thermally stable bioflocculants have been reported, for example, *Bacillus licheniformis* [10], *Bacillus mojavensis* 32A [19], and *Enterobacter cloacae* WD7 [20] keep their flocculation ability below 80°C, 75°C, and 70°C, respectively. ETH-2 bioflocculant had 80.1%flocculation efficiency after being heated for 30 minutes under 100°C, indicating ETH-2 is more thermally stable.

#### Flocculation mechanism

Flocculation occurs through either bridging or charge neutralization and even by a combination of these two mechanisms [1], [40]. Bridging occurs when bioflocculants extend from the particles' surface into the solution for a distance greater than the distance over which the inter-particle repulsion acts and bioflocculants adsorb other particles to form flocs [1]. Charge neutralization occurs when bioflocculant is oppositely charged compared to the particles. Charge is neutralized and the repulsion between particles is eliminated [1]. The zeta potentials of bioflocculant and kaolin clay suspension were −28.7±8.23 and −35.6±1.66, respectively. If charge neutralization was the main mechanism for the flocculation, flocculation should occur when the zeta potential of the particles is sufficiently low to eliminate repulsion between them. However, the zeta potential of the mixture of ETH-2 and kaolin clay retains a large negative value, −46.9±6.72 mV, suggesting that bridging, instead of the charge neutralization, is probably the flocculation mechanism for ETH-2 bioflocculant.

**Figure 10:**
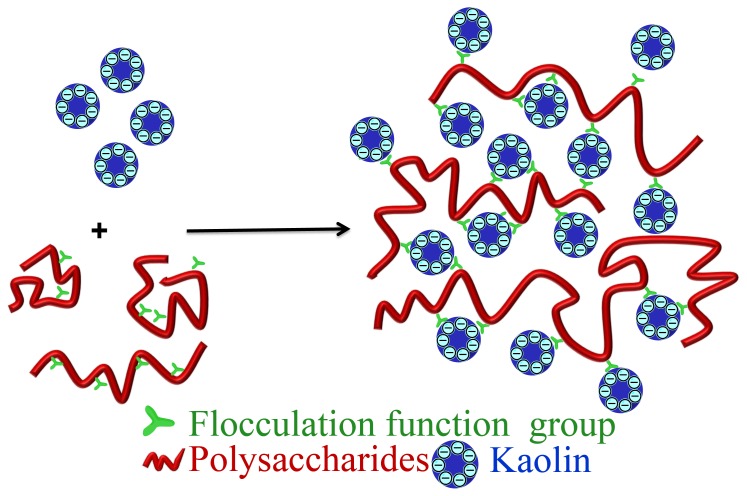
Schematic illustration for the flocculation mechanisms of ETH-2 with Kaolin.

The bridging mechanism depends on the structure of bioflocculants such as chemicals constitution, functional groups and the molecular weight. Polysaccharides produced by *Bacillus megaterium* TF10 have along “backbone” with a large number of active sites that adsorb particles through the bridging mechanism [26]. Polysaccharides produced by *Proteus mirabilis* TJ-1 [3] share the same mechanism. Charge neutralization also acts as the flocculation mechanism for these two bioflocculants. In this study, high MWs polysaccharides in ETH-2 had a long chain structure with small branches. The main backbone of the polysaccharides was composed of mannose, glucose, and galactose. Similar to the structure of polysaccharides produced by *Bacillus megaterium* TF10 [26], the long backbone of polysaccharides with a large number of active sites is believed to be the main reason for the high flocculation activity of the ETH-2. ETH-2 long backbones simultaneously adsorb many particles through large numbers of active sites, forming large flocs (Figure 10). Unlike bioflocculants produced by *Bacillus megaterium* TF10 and *Proteus mirabilis* TJ-1, cation could not improve the flocculation efficiency of ETH-2, indicating that charge neutralization was not the flocculation mechanism. Bridging mechanism then, was concluded as the main flocculation mechanism in this study.

### Conclusion

In summary, bioflocculant produced by the newly isolated microorganism *Entercbacter* sp. ETH-2 has high flocculating activity with unique characters such as cation independence, pH tolerance, and thermal stability. Polysaccharides in the bioflocculant were identified as the active constituents, and the long backbone structure with active sites and high MW were recognized to be responsible for the high flocculation activity through bridging mechanism.

## Supporting Information

File S1 **Combined file of supporting figures and tables.** Figure S1, Optimization of carbon and nitrogen resource for strain ETH-2 growth. Table S1, Comparison of bioflocculants extraction methods. Table S2, Apparent molecular weight data from dextran standards and RI detector. Table S3, Binding energies (eV) and assignment/quantization of XPS spectral bands. Materials S1, Carbon and nitrogen source experiments.
(RAR)
